# Progesterone for the prevention of preterm birth in women with multiple pregnancies: the AMPHIA trial

**DOI:** 10.1186/1471-2393-7-7

**Published:** 2007-06-19

**Authors:** Arianne C Lim, Kitty WM Bloemenkamp, Kees Boer, Johannes J Duvekot, Jan Jaap HM Erwich, Tom HM Hasaart, Pieter Hummel, Ben WJ Mol, Jos PM Offermans, Charlotte M van Oirschot, Job G Santema, Hubertina CJ Scheepers, Willem A Schöls, Frank PHA Vandenbussche, Maurice GAJ Wouters, Hein W Bruinse, the AMPHIA study group

**Affiliations:** 1 Department of Obstetrics and Gynaecology, Academic Medical Centre, Amsterdam, the Netherlands; 2 Department of Obstetrics and Gynaecology, Leiden University Medical Centre, Leiden, the Netherlands; 3 Department of Obstetrics and Gynaecology, Erasmus Medical Centre, Rotterdam, the Netherlands; 4 Department of Obstetrics and Gynaecology, University Medical Centre, Groningen, the Netherlands; 5 Department of Obstetrics and Gynaecology, Catharina Hospital, Eindhoven, the Netherlands; 6 Department of Obstetrics and Gynaecology, Medical Centre Alkmaar, Alkmaar, the Netherlands; 7 Department of Obstetrics and Gynaecology, Máxima Medical Centre, Veldhoven, the Netherlands; 8 Department of Obstetrics and Gynaecology, Academic Hospital Maastricht, Maastricht, the Netherlands; 9 Department of Obstetrics and Gynaecology, St Elisabeth Hospital, Tilburg, the Netherlands; 10 Department of Obstetrics and Gynaecology, Medical Centre Leeuwarden, Leeuwarden, the Netherlands; 11 Department of Obstetrics and Gynaecology, University Medical Centre St Radboud, Nijmegen, the Netherlands; 12 Department of Obstetrics and Gynaecology, Meander Medical Centre, Amersfoort, the Netherlands; 13 Department of Obstetrics and Gynaecology, VU Medical Centre, Amsterdam, the Netherlands; 14 Department of Obstetrics and Gynaecology, University Medical Centre, Utrecht, the Netherlands

## Abstract

**Background:**

15% of multiple pregnancies ends in a preterm delivery, which can lead to mortality and severe long term neonatal morbidity. At present, no generally accepted strategy for the prevention of preterm birth in multiple pregnancies exists. Prophylactic administration of 17-alpha hydroxyprogesterone caproate (17OHPC) has proven to be effective in the prevention of preterm birth in women with singleton pregnancies with a previous preterm delivery. At present, there are no data on the effectiveness of progesterone in the prevention of preterm birth in multiple pregnancies.

**Methods/Design:**

We aim to investigate the hypothesis that 17OHPC will reduce the incidence of the composite neonatal morbidity of neonates by reducing the early preterm birth rate in multiple pregnancies. Women with a multiple pregnancy at a gestational age between 15 and 20 weeks of gestation will be entered in a placebo-controlled, double blinded randomised study comparing weekly 250 mg 17OHPC intramuscular injections from 16–20 weeks up to 36 weeks of gestation versus placebo. At study entry, cervical length will be measured. The primary outcome is composite bad neonatal condition (perinatal death or severe morbidity). Secondary outcome measures are time to delivery, preterm birth rate before 32 and 37 weeks, days of admission in neonatal intensive care unit, maternal morbidity, maternal admission days for preterm labour and costs. We need to include 660 women to indicate a reduction in bad neonatal outcome from 15% to 8%. Analysis will be by intention to treat. We will also analyse whether the treatment effect is dependent on cervical length.

**Discussion:**

This trial will provide evidence as to whether or not 17OHPC-treatment is an effective means of preventing bad neonatal outcome due to preterm birth in multiple pregnancies.

**Trial registration:**

Current Controlled Trials ISRCTN40512715

## Background

Despite the advances in neonatal care during the last decades, preterm birth remains the major cause of handicaps in children without congenital anomalies. Prevention of preterm birth is therefore a major goal of obstetric care. However, strategies to prevent preterm birth have been largely unsuccessful. Recently, it has been shown that prophylactic progesterone administration in women with a singleton pregnancy and a previous preterm birth significantly reduces the incidence of preterm birth [1,2].

### Incidence and financial impact of preterm birth

In women with singleton pregnancies, only 1% delivers prior to 32 weeks. However, after previous preterm delivery this risk increases to 15% [3]. In both asymptomatic singleton and twin pregnancies, a short cervical length is predictive of preterm birth [4]. Multiple pregnancies are by nature at high risk for preterm delivery. In the Netherlands approximately 15% of women with a multiple pregnancy delivers before 34 weeks of gestation [5-7]. At present, approximately 1 in 60 pregnancies is a twin pregnancy, and around 30% of all preterm born children admitted to a neonatal intensive care unit (NICU) are from twin pregnancies [6,7]. The prevalence of twin pregnancies is still rising, partly due to an increase in age of pregnant women and an increase in assisted reproductive technologies. The financial burden of preterm born babies is substantial: about € 1000, – per day when a child is admitted to a NICU with the concomitant costs for both parents and the society in general, in case the child is handicapped.

### Neonatal risks of preterm birth

Bad neonatal outcome includes respiratory distress syndrome (RDS), bronchopulmonary dysplasia (BPD), intraventricular haemorrhage (IVH), periventricular leucomalacia (PVL), necrotizing enterocolitis (NEC), sepsis and death before discharge [8]. The prevalence of this poor neonatal outcome is 77%, 35% and 12% in children born after early preterm delivery between 24–27, 28–32 and 32–34 weeks, respectively [7]. After 34 weeks this incidence sharply declines to less than 2% at term. The probability that a woman with a multiple pregnancy delivers at these gestational ages is 1.8%, 5.4% and 7.2%, respectively [6]. Overall, this means that about 8% of all multiple pregnancies will result in the death of at least one child, whereas in 7% of these pregnancies at least one of the children will remain severely disabled. Moreover, another 20% of the pregnancies will result in a moderate handicap of at least one of the children.

### Progesterone treatment for the prevention of preterm birth

Although preterm birth is known to be the most important complication of multiple pregnancies, no commonly accepted strategy is available to prevent this condition. Progesterone administration (with a recently proven effect on the occurrence of preterm birth in high risk singleton pregnancies [1,2,9]) is not as yet used. In conclusion, no generally established strategy is at present available to prevent the most common and important complication of multiple pregnancies.

We propose an intervention with weekly progesterone injections (250 mg 17 alpha hydroxyprogesterone caproate (17OHPC)) from 16–20 weeks up to 36 weeks of gestation. This intervention had been chosen for two reasons. First, it has been proven that this prophylactic administration of 17OHPC injections is effective in reducing the preterm birth rate in singleton pregnancies at high risk for a spontaneous preterm delivery. Second, a multiple pregnancy is considered to be a pregnancy at high risk for a spontaneous early preterm delivery with a high composite neonatal morbidity rate, as demonstrated by the data shown above.

We choose to use intramuscular administration of progesterone over vaginal application, as we think that compliance can be measured more accurately with the former method.

### Safety of 17-alpha hydroxyprogesterone

In view of the disaster with diethylstilbestrol, it is understandable that many patients, doctors and others are concerned about negative long term side effects on women or their offspring [10]. 17-alpha-hydroxyprogesterone caproate is a natural metabolite of progesterone that is produced by the placenta itself. Apart from a painful spot at the injection side, no side effects have been described regarding the mother or child.

In the last 40 years progestins have been administered to pregnant women for several reasons, including threatening miscarriage, recurrent miscarriage, threatening abortion, prevention of preterm labour and luteal support during IVF treatment. Especially for progestins with an androgenic effect, there was fear for masculinisation of the female foetus. Research among 2500 exposed women and controls showed no difference with respect to the occurrence of abnormalities of the central nerve system, limbs and joints, urogenital tract and circulatory tract between treated and untreated pregnancies, even when 17OHPC was administered in early pregnancy [11-13]. Also, long term follow-up of prenatally exposed young adolescents found no differences from controls with respect to growth, onset of puberty, spatial and verbal test results and sex-dimorphic behaviour and traits [14-16].

## Methods/Design

### Aims

The aim of this study is to investigate whether prophylactic administration of 17OHPC will lower the incidence of neonatal morbidity by reducing the number of preterm births.

We will use a randomised, placebo-controlled double blinded trial (the AMPHIA-trial: 17-Alpha hydroxyprogesterone in Multiple pregnancies to Prevent Handicapped InfAnts) to assess the effects of 17OHPC injections on neonatal outcome. The study is set in the Dutch Obstetric Research Consortium, a collaboration of obstetric practices in the Netherlands. Approximately 50 clinics, including academic hospitals, non-academic teaching hospitals and non-teaching hospitals will participate in this trial.

### Participants/Eligibility criteria

All women with a multiple pregnancy, both mono- and multichorionic, and a gestational age ≤ 19 weeks are eligible for participation in the AMPHIA-trial. Before inclusion, chorionicity must be accurately determined by means of ultrasound.

Women with a previous spontaneous preterm birth < 34 weeks, serious congenital defects or death of one or more foetuses, early signs of twin-to-twin transfusion syndrome or primary cerclage are excluded from the study.

### Procedures, recruitment, randomisation and collection of baseline data

All women with a multiple pregnancy who present at one of the participating clinics will be referred to an obstetrician or a specifically appointed research nurse/midwife for counselling. Eligible women will receive an information sheet and, where possible, are given two weeks time to reflect on participation. Once women have given consent for the trial, they are randomised through a website, according to a computer-generated randomisation sequence. Stratification will be applied for chorionicity (mono- versus multichorionic), number of multiples (twin versus higher order multiple) and previous vaginal birth (nullipara versus multipara). Randomisation will be 1:1 for progesterone and placebo.

When a patient is randomised, the company that supplies the study medication will receive an automatic notification by e-mail. A medication package with the corresponding randomisation number, containing 20 ampoules of 1 cc of either 250 milligrams 17OHPC or placebo, is then sent to the pharmacist in the clinic where the patient has been randomised. After a maximum of 7 days, the medication package will be available to the obstetrician or research nurse/midwife. Content of the package is blinded to the practitioner, the patient and the local pharmacist. In case of severe side-effects, allocation can be disclosed by coded envelopes that are available to the principal investigator.

Baseline demographic, past obstetric and medical histories will be recorded for all women. Cervical length will be measured by transvaginal ultrasound at the time of randomisation or at the next visit.

### Intervention

The patient will receive the first intramuscular injection of 250 milligrams of 17OHPC or placebo at a gestational age between 16^+0 ^and 20^+0 ^weeks. For the remaining duration of the pregnancy, the medication package will be stored by the patient herself. Weekly injections will be administered by a nurse at the outpatient clinic, a general practitioner or the patient herself. Dates of injection are noted in a schedule that is kept both by the patient and in her medical record. Non-compliance is defined as an interval of more than ten days between two injections. Injections are continued weekly until a gestational age of 36 weeks or, in case of delivery before 36 weeks, until delivery. Management of the pregnancy is done according to the local protocol.

### Follow up of women and infants

The patient will be invited for a final visit 6 weeks after delivery. At this visit, all medication that has not been used must be returned and the patient is asked about the condition of her children.

All details of delivery, maternal assessments and admittance during pregnancy are recorded in the case record form that is accessible through the website. In case of admittance of one or more children to the neonatal intensive care unit, details of this admittance are also recorded.

Long-term follow up of children is desirable, but is depending on future funding.

### Outcome measures

The primary outcome measure is composite neonatal morbidity. This composite morbidity rate contains severe RDS, BPD, IVH grade II B or worse, NEC, proven sepsis and death before discharge from the hospital [8].

Secondary outcome measures are time until delivery, preterm birth before 32 and 37 weeks, length of admission at the neonatal intensive care unit, maternal morbidity, hospitalization of the mother due to (threatened) preterm labour and costs.

Analysis is performed according to the intention-to-treat principle. The effectiveness of progesterone compared with placebo will be assessed by calculating a relative risk and 95% confidence interval. Time to delivery will be assessed with Kaplan-Meier analysis and Cox proportional hazard analysis. We will also assess whether there is an interaction between treatment effect and cervical length at 20 weeks.

### Economic evaluation

#### General considerations

The aim of the economic evaluation is to compare the optimality, in terms of costs and health effects, of weekly (prophylactic) administration of 17OHPC progesterone injections (16–20 to 36 weeks) with no intervention. As the clinical study is based on a superiority design (it is hypothesized that progesterone decreases preterm birth considerably), the primary economic evaluation is a cost-effectiveness analysis (CEA): the optimal strategy is the one with the largest health gain at the smallest extra costs.

Costs and outcomes are analysed according to intention-to-treat and described with appropriate statistical measures. The sensitivity of costs and health outcomes for various parameters is tested in sensitivity analysis. Scenario analyses for relevant subgroups (e.g. gestational age at birth, parity, monochorionic/dichorionic multiples) are added.

#### Cost analysis

The process of care is distinguished into three cost stages (antenatal stage, delivery/childbirth, postnatal stage) and three cost categories (direct medical costs [all costs in the health care sector], direct non-medical costs [costs outside the health care sector that are affected by health status or health care] and indirect costs [costs of sick leave]). For each stage and each cost category, costs are measured as the volumes of resources used multiplied with appropriate valuations (cost-per-unit estimates, fees, national reference prices).

Cost volumes in the antenatal stage consist of direct medical costs (e.g. hospital care, outpatient visits, administration of 17OHPC progesterone injections). Direct non-medical and indirect costs in that stage may occur if role patterns or household routines shift.

Costs during childbirth are dominated by the course of childbirth and type of delivery. Cost volumes in the postnatal stage consist of hospital-based maternal care (hospitalization etc.), neonatal care (admission to NICU/medium care or maternity ward, related diagnostic care and treatment, outpatient visits) and primary care. If neonatal health is suboptimal, further direct medical, direct non-medical and indirect costs may occur. Hence, for these infants, resource use of infants and/or parents is measured during 12 months after childbirth.

Volumes of health care resource use are measured prospectively alongside the clinical study in all participating centres as part of the CRF.

Valuations of direct medical resources are estimated as cost per unit estimates comprising "true economic" costs, i.e. including shares of fixed costs and hospital overheads. Costs per units are estimated for at least teaching and one non-teaching hospital. An analysis based on reimbursement fees is added. Direct medical volumes outside the hospital and direct non-medical volumes are valued using national reference prices [17]. Indirect costs are quantified but remain unvalued. Study-specific costs are excluded from analysis.

### Statistical issues

#### Sample size

The sample size is calculated based on the primary outcome 'bad neonatal outcome'. In the control group, 'bad neonatal outcome' is expected in 7.2% of the children (1.8%*77% + 5.4%*35% + 7.2%*12% + 35.6%*8% + 50%*.5% = 7.2%). In this calculation, the first rate represents the probability that a patient delivers at that gestational age, whereas the second rate represents the probability of 'bad neonatal outcome' at that particular gestational age. In case of treatment, 'bad neonatal outcome' is then expected in 3.9% of the children (0.9%*77% + 2.7%*35% + 3.6%*12% + 17.8%*8% + 75%*.5% = 3.9%).

Because the outcomes in children from multiple pregnancies are to a certain extent non-independent, we adjusted our sample size assuming a correlation of 0.6 for composite neonatal morbidity between two children born from the same pregnancy [18]. Using a two-sided test with an alpha of 0.05 and a power of 0.80 we need 330 women in the control group and 330 in the intervention group.

#### Data analysis

Data will initially be analysed according to the intention to treat method. The main outcome variable, 'bad neonatal outcome', will be assessed by calculating rates in the two groups, relative risks and 95% confidence intervals as well as numbers needed to treat. To evaluate the potential of each of the strategies, we will also perform a par protocol analysis, taking into account only those cases that were treated according to protocol.

Time to delivery will be evaluated by Kaplan-Meier estimates, with account for different durations of gestation at entry, and will be tested with the log rank test. The other secondary outcome measures will be approached similarly to the primary outcome measure. The analysis will be stratified for chorionicity, number of multiples and parity. Furthermore, we will take account of non-independence between children born from the same pregnancy using cluster analysis.

#### Interim analysis

An interim analysis will be performed after the inclusion of 300 women. This analysis will be done by an independent data and safety monitoring committee that will not be aware of the allocation of treatment or placebo when they judge data on effectiveness. In case of severe side-effects, the safety monitoring committee can order to disclose the label of patients with such side effects.

### Ethical considerations

This study has been approved by the ethics committee of the Academic Medical Centre Amsterdam (Ref. No. MEC 05/102) and by the boards of management of all participating hospitals.

## Discussion

In the past, many strategies have been applied to prevent preterm birth in multiple pregnancies, such as bed rest, uterine activity monitoring, prophylactic tocolysis and primary cerclage. However, none of these strategies have been proven to be effective.

Whilst the aetiology of spontaneous preterm birth is multifactorial in both singleton and multiple pregnancies, prophylactic administration of progesterone has recently been shown to reduce preterm birth in women with a singleton pregnancy and a previous preterm delivery. From that perspective, and taking into account the major medical and social implications of preterm birth, research has to be done into the possible beneficial effects of progesterone in multiple pregnancies.

Simultaneously with the AMPHIA-trial, a number of other study groups in different countries have set up trials to investigate the effectiveness of progesterone in preventing preterm birth in multiple pregnancies. These are two trials in the United States, one in Canada, one in Scotland, one in Lebanon and one in Denmark [19-24]. Some of these trials use intramuscular injection, analogously to this trial, whilst others use vaginal application. If the outcome data of these studies are pooled, a more conclusive statement can be made on this matter.

## Abbreviations

17OHPC – 17-alphahydroxyprogesterone caproate

NICU – Neonatal Intensive Care Unit

## Competing interests

The author(s) declare that they have no competing interests.

## Authors' contributions

HWB and BWM were involved in conception and design of the study. ACL, HWB and BWM drafted the manuscript. All authors mentioned in the manuscript are members of the AMPHIA study group. They participated in the design of the study during several meetings and are local investigators in the participating centres. All authors edited the manuscript and read and approved the final manuscript.

## Pre-publication history

The pre-publication history for this paper can be accessed here:


